# Computational and Experimental Characterization of *Mycobacterium marinum* β-Carbonic Anhydrase Inhibitors

**DOI:** 10.1177/11779322261427120

**Published:** 2026-02-26

**Authors:** Niaz Morshed, Md. Selim Reza, Ratul Bhowmik, Ashok Aspatwar

**Affiliations:** 1 Department of Pharmacy, Faculty of Pharmacy, University of Dhaka, Dhaka, Bangladesh; 2 Department of Pharmaceutical Technology, Faculty of Pharmacy, University of Dhaka, Dhaka, Bangladesh; 3 Faculty of Medicine and Health Technology, Tampere University, Tampere, Finland

**Keywords:** β-carbonic anhydrase, *Mycobacterium marinum*, molecular docking, molecular dynamics simulation, structure-activity relationship, in vitro assay

## Abstract

Carbonic anhydrases in *Mycobacterium tuberculosis* are increasingly recognized as promising therapeutic targets in drug-resistant tuberculosis. In this study, a homology model of β-carbonic anhydrase was developed using the closely related *Mycobacterium marinum* sequence as a structural basis. A focused antituberculosis compound library was screened, identifying 2 ligands, F2686-0257 and F1011-1367, with strong binding affinities and distinct interaction patterns. Molecular dynamics simulations more than 100 ns confirmed stable backbones and conserved binding pockets, with F2686-0257 stabilized by aromatic anchoring and F1011-1367 by polar interactions. Structure-activity relationship analysis highlighted rigid aromatic scaffolds, controlled molecular size, and balanced polarity as favorable features. In *M marinum* growth assays, F2686-0257 inhibited bacterial proliferation at 100 µM and enhanced rifampicin activity, whereas F1011-1367 showed weaker inhibition without synergy. The compounds also showed favorable ADMET and drug-likeliness properties. These results support β-carbonic anhydrase as a viable target and provide scaffolds for the rational development of novel antitubercular agents.

## Introduction

Nontuberculous mycobacteria (NTM) represent a diverse group of opportunistic pathogens, and infections caused by NTMs have risen sharply in recent years, particularly in developed countries. This increase is largely attributed to aging populations, the growing prevalence of immunosuppressive disorders, and widespread use of immunosuppressive therapies.^1,2^ Among NTMs, *Mycobacterium marinum* (*M marinum*) is of particular interest due to its close genetic and pathogenic relationship with *Mycobacterium tuberculosis*. The *M marinum* genome spans 6.6 Mb about 1.5 times larger than *M tuberculosis* and shares ~85% amino acid identity across orthologous proteins.^3-5^ This expanded genome is thought to equip *M marinum* with the ability to survive fluctuating environmental conditions and infect a broader host range, from fish and amphibians to humans. *M marinum* typically grows at 28°C to 30°C, and its inability to replicate at 37°C limits infections in humans to cooler skin regions.^6^ Human infections, often termed “fish tank granulomas,” are caused by inoculation through cuts or abrasions after contact with contaminated water or aquatic animals.^7,8^ These infections can range from localized dermal granulomas to more invasive diseases, such as tenosynovitis or osteomyelitis, especially in immunocompromised individuals.^9-11^ Treatment is challenging, frequently requiring prolonged antibiotic courses or even surgical intervention when lesions are persistent.^12^

Because *M marinum* and *M tuberculosis* share many virulence strategies, including evasion of host defenses via protein and lipid virulence factors, inhibition of phagolysosome maturation, and survival within a mycobacteria-containing vacuole, it has become a widely used surrogate model for studying tuberculosis pathogenesis.^13-15^ Its larger genome has allowed *M marinum* to maintain traits of both an intracellular pathogen and an environmentally adaptable bacterium, forming biofilms, persisting in protozoa, and retaining the capacity for environmental survival.^16,17^ Among the genes shared by *M marinum* and *M tuberculosis*, carbonic anhydrases (CAs) are emerging as particularly intriguing targets.^18,19^ CAs are metalloenzymes that catalyze the reversible hydration of CO_2_ to bicarbonate, a fundamental reaction for pH regulation and metabolic adaptation. The *M tuberculosis* genome encodes 3 β-class carbonic anhydrases (β-CAs) and 1 γ-CA, enzymes implicated in acid stress adaptation and virulence.^18,20^ Despite their importance, the structural and mechanistic details of these enzymes remain poorly understood in both *M tuberculosis* and *M marinum*, limiting their potential exploitation as drug targets.

Here, we present an integrative computational and in vitro approach to characterize β-carbonic anhydrase that is obtained from NCBI (WP_020730851.1), which is unique for *M marinum*, and explore its potential as a therapeutic target. Using homology modeling, virtual screening, molecular docking, and molecular dynamics simulations, we identified and validated promising small-molecule inhibitors with strong binding affinities and stable interactions. These molecular studies were further complemented by data-driven structural activity relationship (SAR) analysis and an in vitro study. Together, these findings advance mechanistic understanding of the *M marinum* β-CA and lay the foundation for rational development of β-CA inhibitors, which can be further used to combat drug-resistant tuberculosis.

## Materials and Methods

### Homology modeling

For homology modeling, we selected the β-CA protein sequence from *M marinum* (NCBI RefSeq: WP_020730851.1) as the modeling basis. Homology modeling was performed in SWISS-MODEL^21^ using the target FASTA sequence. The target was first aligned on the server to identify candidate templates. Afterwards the template with the highest sequence identity and GMQE (Global Model Quality Estimate) score was used to build the homology models and exported as PDB for further analysis.

### Virtual screening of antituberculosis compounds against carbonic anhydrase

#### Compound library selection

The objective of the virtual screening was to identify potential inhibitors of carbonic anhydrase with antituberculosis activity. For this purpose, we obtained the focused antituberculosis screening library from the Life Chemicals database (https://lifechemicals.com), which provides a diverse collection of small molecules designed for drug discovery applications. This Screening Library was created through a 2D fingerprint similarity search against a reference set of 23 734 biologically active compounds (IC50, Ki, etc, less than 10 μM, Inhibition > 25%) extracted from the Binding and ChEMBL databases against prominent protein targets and protein families. . From this resource, we selected a focused set of 4254 compounds enriched for activity against carbonic anhydrases. The library was downloaded in the Structure Data File (SDF) format for subsequent filtering and analysis (Supplementary File: 2).

#### Multistep library filtration

Following the selection of the focused library containing 4254 compounds, a multistep filtration strategy was applied prior to molecular docking and molecular dynamics simulations. Initially, physicochemical and drug-likeness filters were applied using the ChemBioServer 2.0 platform (https://chembioserver.vi-seem.eu/index.php).^22^ To enrich for compounds with favorable oral bioavailability and pharmacokinetic properties, we applied Ghose’s filter, Veber’s rules, and Lipinski’s rule of five. This step reduced the library for further evaluation. The filtered library was then subjected to toxicophore analysis to improve the predicted safety profile and remove molecules with potential toxic liabilities. Finally, Pan-Assay Interference Compounds (PAINS) were excluded using the PAINS remover tool (https://www.cbligand.org/PAINS/) eliminating likely false positives.

#### Molecular docking studies

Molecular docking was performed using BIOVIA Discovery Studio 2019 and PyRx.^23^ The homology-modeled β-carbonic anhydrase structure was first prepared in Discovery Studio by removing water molecules and heteroatoms. The ligand library was energy-minimized using the Universal Force Field (UFF) to generate stable, low-energy conformations. The protein was converted into macromolecule format in PyRx, and the screened compounds were designated as ligands. Docking was carried out using the AutoDock Vina module within PyRx. The docking grid was defined to encompass the entire protein, with the following parameters: center_x = 7.2641, center_y = 3.4623, center_z = –2.0304; size_x = 34.6478, size_y = 34.6478, size_z = 57.7113 (all values in Å). An exhaustiveness value of 8 was used to ensure sufficient conformational sampling. Protein-ligand interactions were analyzed and visualized using Discovery Studio.

#### Molecular dynamics simulation studies

The 2 top-performing ligands from docking were subjected to 100 ns molecular dynamics (MD) simulations to evaluate the stability of the protein-ligand complexes. Simulations were carried out in Desmond^24^ using the OPLS3e force field.^25^ Each complex was placed in a 10 Å × 10 Å × 10 Å orthorhombic simulation box, solvated with an isotonic saline solution. Counterions (Na^+^; or Cl^−^) were added as needed to neutralize the system. Molecular dynamics simulations were performed under NPT ensemble conditions at 300 K and 1.02325 bar.^26^ Trajectories were recorded for 1000 frames more than 100 ns, with snapshots saved every 100 ps for subsequent analysis.

#### Structural activity relationship analysis

To gain insights into the detailed structural activity relationship (SAR) of our docked molecules, we further calculated a total of 307 substructure fingerprints as well as 125 physicochemical descriptors using PaDEL software (http://www.yapcwsoft.com/dd/padeldescriptor/)^27^ for all the molecules. We further labeled the molecules into 3 different classes based on docking scores. Molecules with a docking score (binding affinity) greater than or equal to −8 kcal/mol were classified as “high binding inhibitors”; molecules having a docking score in the range of −6 to 8 kcal/mol were classified as “average binding inhibitors”; whereas molecules having a binding affinity less than −6 kcal/mol were classified as “low binding inhibitors.” For a descriptive statistical analysis, we computed Pearson’s correlation coefficient between each descriptor/fingerprint and each activity class. A correlation matrix was further constructed, where rows represented activity classes and columns presented molecular features (physicochemical descriptors/molecular fingerprints). This further quantified the linear association of each molecular feature with each activity class (based on binding affinities), thereby enabling feature relevance evaluation. The maximum absolute correlation values across activity classes were further computed to identify molecular features with the strongest discriminatory potential. Based on these scores, the top-ranked 30 molecular features (separately for substructure fingerprints and physicochemical descriptors) were selected for downstream visualization through a Pearson correlation plot analysis.

#### ADME, toxicity profile, and drug-likeness predictions

The pharmacokinetic, drug-likeness, and toxicity profiles of the selected compounds (F2686-0257 and F1011-1367) were evaluated using web-based computational tools. Canonical SMILES of the compounds were submitted to the SwissADME server (http://www.swissadme.ch/index.php)^28^ to predict ADME and lead likeness profile. Toxicity class and LD50 values were predicted using the ProTox-II server (http://tox.charite.de/), where compounds were classified according to the globally harmonized system (GHS).^29^

#### Validating computationally predicted hits via in vitro growth inhibition of *Mycobacterium marinum*

The best-performing inhibitors, identified through the machine learning–driven cheminformatics pipeline, were purchased from Life Chemicals, Inc. (Burlington, Ontario, Canada; https://lifechemicals.com/). The compounds were dissolved in DMSO and distilled water to prepare 100 mM stock solutions. A series of dilutions for each compound was then prepared in ddH_2_O before performing inhibition assays against *M marinum* in 96-well plates. The screening was carried out following a previously described method with slight modifications.^30^ A bioluminescence-cassette (*pMV306hsp* *+* *LuxG13*) expressing *M marinum* (ATCC 927, Mmr) strain was used for the inhibition studies. The *pMV306hsp* *+* *LuxG13* plasmid was a kind gift from Brian Robertson and Siouxsie Wiles (Addgene plasmid #26161; http://n2t.net/addgene:26161; RRID: Addgene_26161).^31^

The bacteria were cultured on Middlebrook 7H10 agar plates (Becton, Dickinson and Company, Franklin Lakes, New Jersey) supplemented with 10% (v/v) oleic acid-dextrose-catalase (BD BBL Middlebrook OADC Enrichment, Becton, Dickinson and Company) and 0.5% (v/v) glycerol at 29°C in the dark for 7 days. For biofilm formation, the bacteria were inoculated into Middlebrook 7H9 broth (Sigma-Aldrich, Missouri) supplemented with 10% (v/v) albumin-dextrose-catalase (BD BBL Middlebrook ADC Enrichment, Becton, Dickinson and Company). Bacterial suspensions were adjusted to an OD_600_ of 0.1 and distributed into sterile white 96-well plates (PerkinElmer, Waltham, Massachusetts) at 184 µL per well. The plates were sealed with Parafilm M (Bemis) and incubated in the dark at 29°C for 7 days.

## Results

### Homology modeling

**Figure 1.**
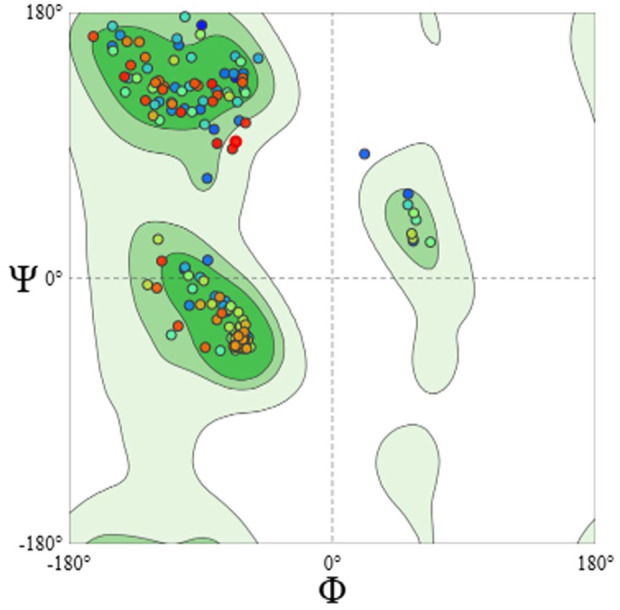
Ramachandran plot of the Homology Model showcasing most residues in favorable region.

For the homology model the selected template was AlphaFold^32^ DB entry A0A2Z5YMS5_MYCMR, which corresponds to a carbonic anhydrase family protein from *M marinum*. This template showed 100% sequence identity to the target input reference sequence (NCBI RefSeq: WP_020730851.1) and a GMQE of 0.89 suggesting high sequence similarity and adequate structural coverage with high confidence. In the quality assessment of the model, structural evaluation metrics confirmed its stereochemical correctness and overall geometric reliability. 96.54% of residues lie in the Ramachandran favored regions indicating a high fraction of conformationally acceptable backbone dihedrals. This level of Ramachandran favorability is consistent with a well-refined homology model and further supported its validity for downstream computational studies (eg, docking, molecular dynamics) (Figure 1).

### Multistep library filtration

**Figure 2.**
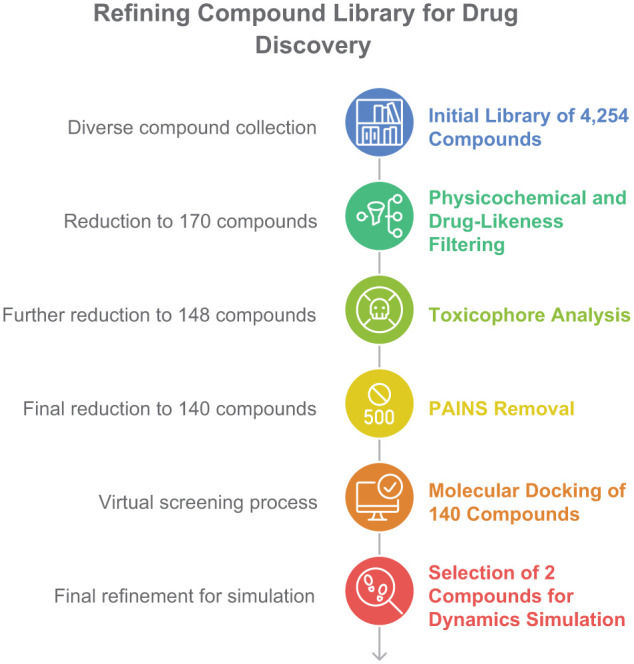
Multistep filtration strategy for the compounds.

Beginning with an initial set of 4254 molecules, drug-likeness filters were first applied, which reduced the library to 170 compounds with favorable properties. Subsequent toxicophore analysis further narrowed this pool to 148 compounds by excluding those with potential toxicity risks. Finally, PAINS (Pan-Assay Interference Compounds) removal was conducted to eliminate likely false positives, resulting in a final selection of 140 compounds suitable for downstream screening (Figure 2). This systematic filtration progressively enriched the library for chemical entities with optimal drug-like characteristics and minimized safety concerns. These final 140 compounds were docked with the homology modeled β-carbonic anhydrase for further studies.

### Binding affinities and ligand interactions

**Figure 3.**
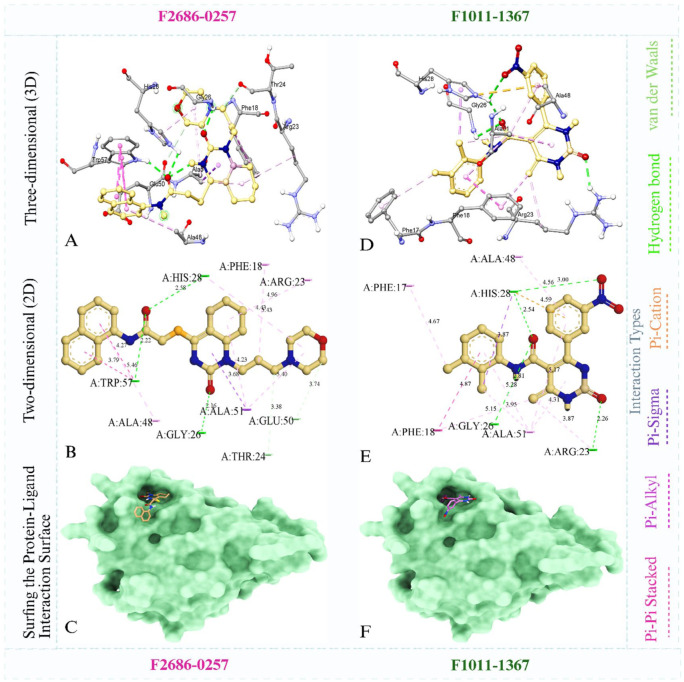
Docking pose of protein and ligand complex file while images (A-C) show for compound F2686-0257, and images (D-F) show for compound F1011-1367.

All screened 140 compounds were docked against the modeled protein, yielding binding affinities from −9.6 to −5.6 kcal/mol (Supplementary File 1). The 2 best-scoring compounds, F2686-0257 (−9.6 kcal/mol) and F1011-1367 (−9.4 kcal/mol), were prioritized because they fell within the highest-affinity region of the distribution (⩽ −9.0 kcal/mol) and thus represent the most promising candidates for further optimization. This practice of retaining only a few top-ranked, high-affinity docking hits is standard in virtual screening to enrich streamline subsequent experiments. The top 2 compounds F2686-0257 and F1011-1367 were further analyzed for detailed interactions with the modeled β-carbonic anhydrase. Compound F2686-0257 exhibited the strongest binding affinity (−9.6 kcal/mol). This ligand formed a π-π stacking interaction between its naphthalene moiety and Trp57, while the same residue also established a hydrogen bond with the oxygen atom of the formamide group. His28 participated in additional hydrogen bonding with the formamide oxygen, and Gly26 interacted through hydrogen bonding with the oxygen atom of the hexahydroquinazolin-2(1H)-one moiety. Thr24 and Glu50 contributed van der Waals contacts, while Ala51 engaged in a π-σ interaction with the hexahydroquinazolin-2(1H)-one ring (Figure 3). Furthermore, Phe18, Arg23, His28, Ala51, and Ala48 established π-alkyl interactions. Compound F1011-1367 also demonstrated strong binding affinity (−9.4 kcal/mol). It formed a hydrogen bond between the oxygen atom of its 3,4-dihydropyrimidinone group and Arg23, while Gly26 and His28 each formed hydrogen bonds with the oxygen atom of the formamide group. An additional hydrogen bond was observed between His28 and the nitrobenzene oxygen (Figure 3). His28 also displayed a π-σ interaction, while Phe18, Ala48, and Ala51 contributed π-π stacking contacts. Phe17, Ala51, and Phe18 established π-alkyl interactions, and Phe28 formed a π-cation interaction.

### Molecular dynamics simulation studies

**Figure 4.**
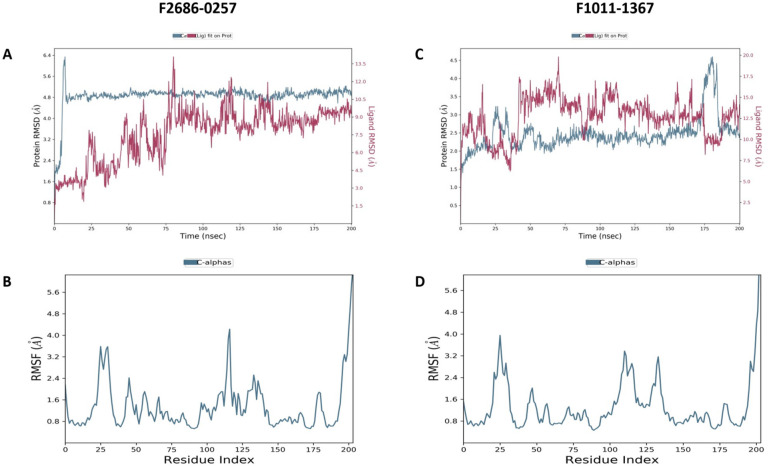
RMSD and RMSF plots of molecular dynamics (MD) of complexes of 2 compounds docked with the CA model protein, while images (A-B) show for compound F2686-0257, and images (C-D) show for compound F1011-1367.

One hundred nanoseconds of simulation were completed for the 2 β-carbonic anhydrase complexes with an equilibration period during the first 15 to 20 ns. After this phase the protein backbone remained stable in both systems. The backbone of the F2686-0257 complex remained close to 4.8-5.2 Å, while the F1011-1367 complex stabilized between 2.0 and 2.5 Å, showing that the overall protein fold was maintained. Ligand RMSD values were higher than the backbone, with F2686-0257 fluctuating mainly between 6 and 14.3 Å and F1011-1367 sampling a wider range between 10 and 19 Å. Average ligand RMSD values across the simulations were 1.81 ± 0.80 Å for F2686-0257 and 1.77 ± 0.13 Å for F1011-1367, which reflects a somewhat steadier orientation for F1011-1367 compared with the broader movement of F2686-0257 (Figure 4). RMSF values of the protein remained low in both systems, with averages near 0.89 ± 0.15 Å.

**Figure 5.**
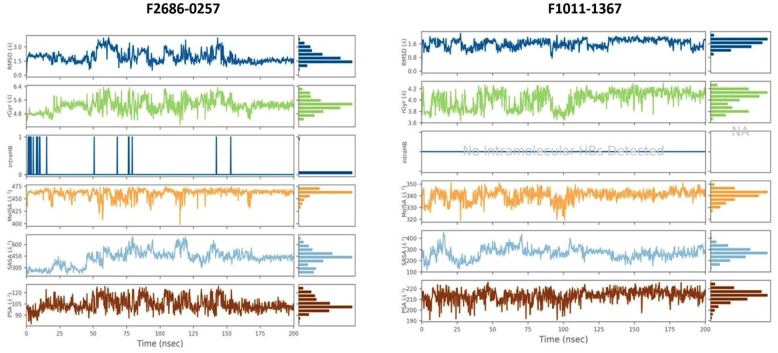
Ligand property plots (RMSD, Radius of Gyration, Intra HB, MoLSA, SASA, and PSA) of molecular dynamics (MD) of protein-ligand complexes of hit compounds F2686-0257 and F1011-1367, respectively.

Global shape and solvent exposure measurements supported these findings. The F1011-1367 complex maintained a smaller radius of gyration at 4.011 ± 0.153 Å and a lower solvent-accessible surface area at 266.844 ± 49.246 Å^2^, whereas the F2686-0257 complex showed a larger radius of gyration at 5.285 ± 0.382 Å and higher solvent exposure at 426.302 ± 99.530 Å^2^ (Figure 5). The radius of gyration values remained stable with low variation, suggesting that secondary structural elements were compactly packed during the simulation. SASA values indicated persistent solvent exposure around the binding pocket, supporting an accessible and flexible site for ligand interaction. RMSF values remained below 1 Å for most residues, consistent with limited conformational changes and overall protein stability. Per-ligand surface descriptors also differed. F1011-1367 exhibited MolSA 340.198 Å^2^ and PSA 213.338 Å^2^ with no intramolecular hydrogen bonds, while F2686-0257 exhibited MolSA 460.846 Å^2^ and PSA 104.463 Å^2^ with a mean intramolecular hydrogen bond count of 0.021 reaching up to 1. Secondary structure analysis showed that both complexes retained the overall fold of the protein. For F2686-0257, the protein maintained 3574% helices and 13.90% strands (total SSE 49.63%). For F1011-1367, helices comprised 32.33% and strands 13.12% (total SSE 45.44%). The differences were modest and indicate no major disruption of the protein architecture during simulation. F2686-0257 formed a hydrogen bond with Gly26 and Glu50 together with π-π contacts to His28 and additional hydrophobic and water-bridge contacts involving Trp57, which helps explain its mid-range ligand RMSD while preserving overall order. F1011-1367 engaged more frequent polar and water-mediated contacts with Phe18, Arg23, and Ser179, including an ionic contact with Arg23, consistent with a compact protein scaffold but a ligand that reorients more often inside a shallow or partially solvent-exposed pocket. Thus, both ligands remained stably associated with the protein, but with distinct dynamic signatures that may represent complementary modes of stabilization suitable for further optimization.

### Structural activity relationship analysis

**Figure 6.**
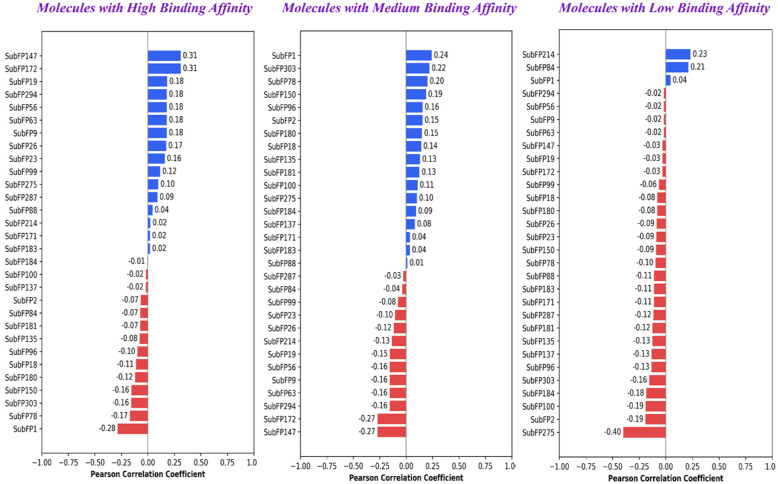
Pearson correlation plot displaying the contribution of the top 30 substructure fingerprints for each activity class.

For substructure fingerprint-guided SAR analysis, we observed positive correlations with respect to molecular features such as urea (SubFP147), arylfluoride (SubFP172), diarylether (SubFP19), and heteroaromatic groups (SubFP184) for high-binding-affinity molecules. These features appear to stabilize binding, likely by providing a rigid framework and hydrophobic or π-stacking interactions within the enzyme pocket. Conversely, structural features such as primary carbon (SubFP1), Michael acceptor groups (SubFP303), thiol derivatives (SubFP78), and isothiourea (SubFP150) correlated negatively with binding affinity, suggesting they might destabilize interactions when present in high-affinity scaffolds. For medium binding affinity molecules, moderate positive correlations were observed for primary carbon (SubFP1), Michael acceptor (SubFP303), isothiourea (SubFP150), and secondary carbon (SubFP2), which suggests that they might act as moderate binders by introducing polarity or flexibility. However, features such as arylfluoride (SubFP172), urea (SubFP147), acetals (SubFP63, SubFP294), and sulfonic derivatives (SubFP214) showed negative correlations, implying that these groups may not favor binding in weaker scaffolds for medium binding affinity molecules. For low-affinity binding affinity molecules, very few features showed positive correlation effects, such as sulfonic derivatives (SubFP214) and carboxylic acid groups (SubFP84), which suggest that these features might provide limited stabilization in terms of binding. Most features, including arylfluoride (SubFP172), urea (SubFP147), thiol derivatives (SubFP78), and heteroaromatic substituents (SubFP184), showed negative correlations (Table 1, Figure 6).

**Table 1.** Pearson correlation scores of top 30 substructure fingerprints with respect to docking scores.

Fingerprints	Interpretation	Average binding	High binding	Low binding
SubFP275	Heterocyclic	0.1021	0.0980	−0.3997
SubFP147	Urea	−0.2719	0.3099	−0.0313
SubFP172	Arylfluoride	−0.2719	0.3099	−0.0313
SubFP1	Primary carbon	0.2421	−0.2842	0.0437
SubFP214	Sulfonic derivative	−0.1347	0.0246	0.2321
SubFP303	Michael acceptor	0.2211	−0.1579	−0.1553
SubFP84	Carboxylic acid	−0.0369	−0.0707	0.2122
SubFP78	Thioenolether	0.2025	−0.1685	−0.0962
SubFP2	Secondary carbon	0.1542	−0.0672	−0.1908
SubFP100	Secondary amide	0.1086	−0.0190	−0.1887
SubFP150	Isothiourea	0.1883	−0.1557	−0.0914
SubFP184	Heteroaromatic	0.0933	−0.0053	−0.1833
SubFP19	Diarylether	−0.1544	0.1830	−0.0313
SubFP9	Alkylfluoride	−0.1558	0.1776	−0.0179
SubFP63	Acetal like	−0.1558	0.1776	−0.0179
SubFP294	Trifluoromethyl	−0.1558	0.1776	−0.0179
SubFP56	Acetal	−0.1558	0.1776	−0.0179
SubFP26	Tertiary aliph amine	−0.1181	0.1725	−0.0864
SubFP23	Amine	−0.1048	0.1595	−0.0889
SubFP96	Carbodithioic ester	0.1560	−0.0987	−0.1338
SubFP180	Hetero N basic no H	0.1503	−0.1213	−0.0787
SubFP18	Alkylarylether	0.1423	−0.1128	−0.0785
SubFP137	Vinylogous ester	0.0828	−0.0202	−0.1330
SubFP135	Vinylogous carbonyl or carboxyl derivative	0.1316	−0.0763	−0.1263
SubFP181	Hetero N nonbasic	0.1257	−0.0708	−0.1245
SubFP99	Primary amide	−0.0764	0.1162	−0.0648
SubFP287	Conjugated double bond	−0.0278	0.0900	−0.1152
SubFP171	Arylchloride	0.0355	0.0192	−0.1105
SubFP183	Hetero S	0.0355	0.0192	−0.1105
SubFP88	Carboxylic acid derivative	0.0120	0.0437	−0.1088

**Figure 7.**
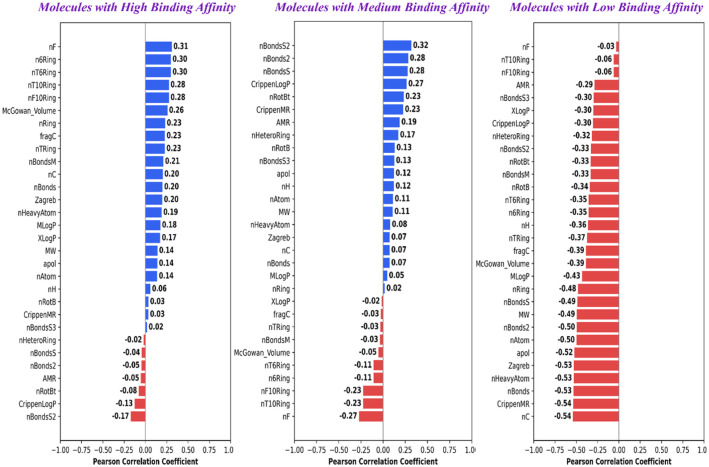
Pearson correlation plot displaying the contribution of the top 30 physicochemical descriptors for each activity class.

For physicochemical descriptor-guided SAR analysis, positive correlations were observed for descriptors such as number of fluorine atoms (nF), 6-, 10-, and 16-membered rings (n6Ring, nT6Ring, nT10Ring, nF10Ring), McGowan volume, ring count (nRing, nTRing), and bond-related descriptors (nBonds, nBondsM) for high binding affinity molecules. The presence of these molecular descriptors suggests that ring systems, molecular size/volume, and controlled fluorination might enhance the stability of binding. Besides these other molecular descriptors, such as XLogP and molecular weight (MW), also showed moderate positive contributions, indicating that hydrophobicity and larger molecular frameworks are favorable within a certain range. Negative correlations were found for CrippenLogP, CrippenMR, AMR, nRotB, nBonds2, nBonds5, nHeteroRing, and nBondsS2, implying that excessive polarity, high flexibility, and certain hetero-ring contributions reduce binding efficiency. For medium binding affinity molecules, positive correlations were observed for descriptors such as bond counts (nBondsS2, nBonds2, nBonds5, nBondsS3), CrippenLogP, rotatable bonds (nRotBt), and AMR, suggesting that these features might moderate flexibility, lipophilicity, and polarizability, and help maintain significant binding affinity. We also observed smaller positive effects for molecular descriptors such as atom count (nAtom), molecular weight (MW), and number of hetero rings (nHeteroRing). Finally, negative correlations were observed for descriptors such as large ring counts (nT10Ring, nF10Ring, nT6Ring, n6Ring), fluorine atoms (nF), and McGowan volume, indicating that excessive size, fluorination, or macrocycles were not favorable for binding. For low-binding-affinity molecules, nearly all molecular descriptors such as ring counts (nRing, nTRing, nT6Ring, n6Ring, nF10Ring, nT10Ring), McGowan volume, molecular weight (MW), atom counts (nAtom, nHeavyAtom), bond counts (nBonds, nBondsM, nBonds2, nBonds5, nBondsS, nBondsS2, nRotBt), and lipophilicity measures (XLogP, MLogP, CrippenLogP), showed strong negative correlations. This further suggests that molecules with high size, flexibility, or excessive hydrophobicity tend to destabilize binding when starting from weak scaffolds (Table 2, Figure 7).

**Table 2.** Pearson correlation scores of top 30 physicochemical descriptors with respect to docking scores.

Molecular descriptors	High binding	Average binding	Low binding
nC	0.2027	0.0726	−0.5398
CrippenMR	0.0344	0.2269	−0.5364
nBonds	0.2002	0.0722	−0.5340
nHeavyAtom	0.1900	0.0800	−0.5307
Zagreb	0.1965	0.0734	−0.5294
apol	0.1394	0.1222	−0.5210
nAtom	0.1385	0.1119	−0.4979
nBonds2	−0.0478	0.2831	−0.4951
MW	0.1408	0.1081	−0.4945
nBondsS	−0.0440	0.2758	−0.4873
nRing	0.2314	0.0179	−0.4814
MLogP	0.1753	0.0463	−0.4326
McGowan_Volume	0.2576	−0.0523	−0.3861
fragC	0.2281	−0.0251	−0.3860
nTRing	0.2266	−0.0294	−0.3743
nH	0.0576	0.1213	−0.3620
n6Ring	0.2989	−0.1059	−0.3543
nT6Ring	0.2989	−0.1059	−0.3543
nRotB	0.0345	0.1320	−0.3398
nBondsM	0.2101	−0.0350	−0.3309
nRotBt	−0.0790	0.2325	−0.3303
nBondsS2	−0.1727	0.3184	−0.3285
nHeteroRing	−0.0212	0.1720	−0.3159
nF	0.3099	−0.2719	−0.0313
CrippenLogP	−0.1283	0.2651	−0.3035
XLogP	0.1729	−0.0153	−0.3003
nBondsS3	0.0183	0.1258	−0.2960
AMR	−0.0534	0.1869	−0.2850
nF10Ring	0.2750	−0.2251	−0.0614
nT10Ring	0.2750	−0.2251	−0.0614

The overall SAR analysis highlights the importance of a rigid, diarylether-linked aromatic core, together with heteroaromatic rings and strategically placed aryl-fluorine groups, for the rational design of potent *M marinum* β-CA4 inhibitors. These structural features were observed to promote rigidity, hydrophobic and π-interactions, and the correct positioning of the zinc-binding group. From the physicochemical descriptor analysis, descriptors such as controlled ring count (nRing, nTRing, n6Ring, nT6Ring), moderate molecular size and volume (MW, McGowan volume), limited fluorination (nF), and balanced hydrophobicity (XLogP, MLogP) were found to support strong binding. In contrast, molecular descriptors linked to excessive polarity or polarizability (AMR, CrippenLogP, CrippenMR), high flexibility (nRotB, nRotBt), macrocyclic or oversized ring counts (nT10Ring, nF10Ring), and large bond counts (nBonds2, nBonds5, nBondsS2) showed negative correlations and were unfavorable for binding. Similarly, substructures such as sulfonyl, urea, acetal-like groups, Michael acceptors, thiols, and flexible linkers showed negative correlation with binding affinity scores. These insights suggest that the most effective β-CA4 inhibitors should have a rigid aromatic scaffold with well-balanced size, limited fluorination, and controlled lipophilicity, while avoiding extra bulk, excessive polarity, and high flexibility.

### ADME, toxicity profile, and drug-likeness predictions

Both compounds, F2686-0257 and F1011-1367, demonstrated favorable pharmacokinetic properties with high GI absorption and moderate oral bioavailability scores (0.55) making them promising candidates for further development. Neither compound showed BBB permeability, which may reduce the risk of central nervous system-related side effects. The predicted skin permeabilities indicate comparable dermal absorption, supporting their potential versatility in administration routes (Table 3).

**Table 3.** ADME properties of 2 compounds.

Compound	GI absorption	BBB permeant	Pgp substrate	log Kp (cm/s)	Bioavailability score
F2686-0257	High	No	Yes	−6.61	0.55
F1011-1367	High	No	Yes	−6.81	0.55

In terms of drug-likeness, both molecules showed strong compliance with Lipinski, Veber, Egan, and Muegge filters, with no violations recorded. F1011-1367 exhibited a particularly clean profile, with no Ghose violations and only a single lead-likeness violation, suggesting an advantage in early drug discovery pipelines. Meanwhile, F2686-0257, despite 2 Ghose violations and more lead-likeness alerts, still retained an overall drug-like profile. These results suggest that both compounds hold promise and have a good balance of drug-likeness parameters (Table 4).

**Table 4.** Drug likeness of the top 2 compounds.

Compounds	Lipinski #violations	Ghose #violations	Veber #violations	Egan #violations	Muegge #violations
F2686-0257	0	2	0	0	0
F1011-1367	0	0	0	0	0

F2686-0257 and F1011-1367 falls under the toxicity class of 4, which is defined as “harmful if swallowed.” But the predicted LD50 value for F2686-0257 is 883 mg/kg where for F1011-1367 it is 1000 mg/kg (Table 5). This high LD50 values suggest low toxicity profiles for both of the compounds.

**Table 5.** Toxicity class and LD50 values of the top 2 compounds.

Compounds	Toxicity class	Predicted LD50 (mg/kg)
F2686-0257	4	883
F1011-1367	4	1000

### Validation of computational hits by in vitro inhibition of *Mycobacterium marinum*

**Figure 8.**
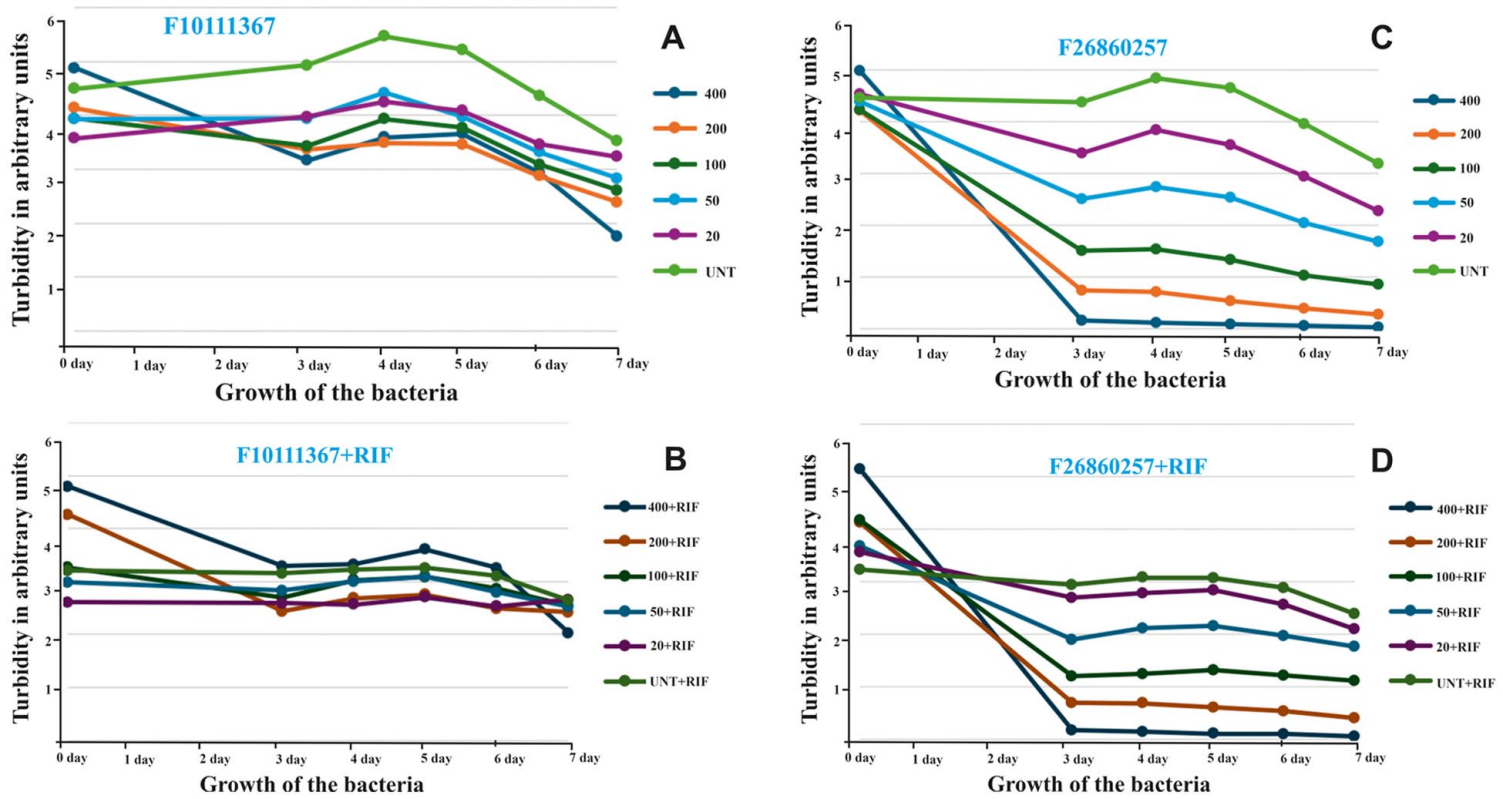
Inhibition of *M marinum* in culture by the identified compounds and/or rifampicin. Panel A shows the growth inhibition of *M marinum* by compound F10111367, while Panel B depicts its effect in combination with rifampicin. Panel C illustrates the inhibition of *M marinum* by compound F26860257, and Panel D shows the synergistic effect when F26860257 is combined with rifampicin. Growth inhibition was evaluated across multiple compound concentrations over time; MIC values were not formally determined.

We next sought to determine whether the identified computational hits, F10111367 and F26860257, inhibit the growth of *M marinum* in vitro. Standard inhibition studies were performed using liquid cultures of *M marinum* in 96-well plates to validate our computational predictions. In addition to visual inspection, culture luminescence was measured over a 0- to 7-day period. In preliminary experiments, a concentration range of 25 to 400 µM was tested. Compound F10111367 showed no reduction in bacterial growth at 3 days post-treatment; however, a reduction in growth was observed at 7 days post-treatment at 400 µM (Figure 8A). When F10111367 was combined with rifampicin, no synergistic effect on bacterial growth was observed (Figure 8B).

In contrast, compound F26860257 demonstrated a marked reduction in bacterial growth at 100 µM as early as 3 days post-treatment (Figure 8C). Furthermore, F26860257 exhibited a synergistic effect when combined with rifampicin (Figure 8D). Thus, this compound not only inhibited bacterial growth on its own but also enhanced rifampicin’s activity (Figures 8C and 8D). These experiments were designed to assess concentration- and time-dependent growth inhibition and drug-interaction trends rather than to determine formal minimum inhibitory concentration (MIC) values using standardized broth microdilution protocols.

### Comparative analysis of the hit compounds

**Figure 9.**
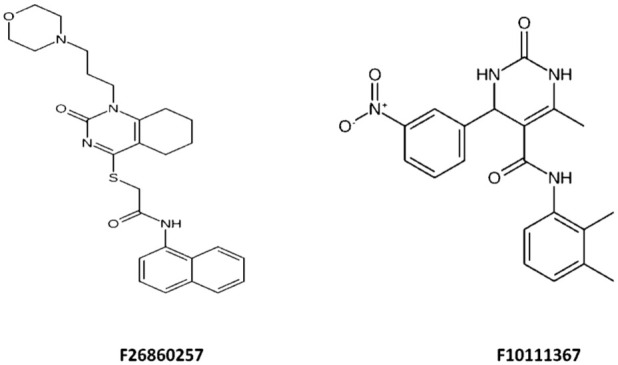
Structural features of the 2 compounds.

The structures of F26860257 and F10111367 (Figure 9) highlight how differences in chemical scaffolds and functional group composition are reflected across docking, dynamics, SAR, ADMET, and in vitro outcomes. Docking indicated that F26860257 has a rigid polyaromatic scaffold incorporating a naphthalene moiety linked to a heterocyclic core bearing a formamide occupies the β-carbonic anhydrase binding pocket through π-π stacking with Trp57 together with complementary hydrogen bonding involving His28 and Gly26. F1011-1367 is built on a more polar scaffold comprising a dihydropyrimidinone core, a formamide linker, and a nitro-substituted aromatic ring and its docked pose is stabilized by hydrogen bonding and polar interactions with residues such as Arg23 and His28. These interaction patterns were maintained during MD simulations, where F2686-0257 showed stabilization through persistent aromatic anchoring and hydrophobic packing despite moderate ligand mobility, whereas F1011-1367 exhibited greater ligand reorientation within a more compact but partially solvent-exposed pocket, consistent with its higher polarity and reduced aromatic surface area. These binding modes are further consistent with SAR trends derived from substructure fingerprint and physicochemical descriptor analyses, which associate higher binding affinity with rigid aromatic and heteroaromatic systems, controlled ring counts and balanced molecular size and polarity, while identifying excessive flexibility, high rotatable bond counts, and strongly polar or reactive functional groups as unfavorable. Both compounds conform to these broader SAR patterns in different ways and retain acceptable predicted ADMET profiles, including high gastrointestinal absorption, compliance with major drug-likeness filters and low predicted acute toxicity. In vitro *M marinum* growth assays further demonstrated that both scaffolds translate computationally defined interaction features into measurable cellular effects. The integrated docking, SAR, ADMET, and biological data provide a coherent medicinal chemistry framework linking scaffold architecture and functional group selection to β-carbonic anhydrase engagement and mycobacterial growth inhibition.

## Discussion

The objective was to identify carbonic anhydrase-focused chemotypes with antituberculosis potential and to understand how they engage a modeled β-carbonic anhydrase. Screening and filtering delivered 2 leads with clear and different interaction patterns. Docking showed that F2686-0257 interacts with Trp57 through π-π stacking and forms hydrogen bonds with His28 and Gly26. In contrast, F1011-1367 relied more on polar interactions, including hydrogen bonding with Arg23 and water-mediated links to Phe18 and Ser179. These patterns matched their chemical properties: F2686-0257 has a larger, less polar surface, favoring aromatic and hydrophobic contacts, while F1011-1367 is smaller and more polar, supporting hydrogen bond networks.

Molecular dynamics simulations confirmed these differences. Both ligands slightly stabilized after 15 to 20 ns, and the protein backbone remained stable, showing the pocket was slightly intact. F2686-0257 was slightly stable in its binding pose, though the protein showed higher solvent exposure and a larger radius of gyration. F1011-1367 is bound in a more compact environment, but the ligand itself moved around more, suggesting less stable positioning. Biological assays in *M marinum* supported the computational results. F2686-0257 inhibited bacterial growth at 100 µM within 3 days and increased the effect of rifampicin. F1011-1367 required 400 µM and 7 days to show inhibition, without synergy. This indicates that F2686-0257 is a slightly good candidate. Substructure and property analysis gave further design clues. Active compounds often contained rigid aromatic rings, diarylether linkages, and fluorine substitutions, which improved π stacking and binding precision. Compounds with too many rotatable bonds, high lipophilicity, or reactive groups showed weaker activity. A practical path forward is to increase hydrogen bond capacity around the Gly26 and His28 region for F2686-0257 without losing the π stack to Trp57, and to add carefully placed aromatic surface for F1011-1367 aimed at Trp57 and Phe18 while maintaining its favorable polar network.

Prior studies provide useful context for these observations. Mycobacterial carbonic anhydrases have been cloned, structurally characterized, and linked to key physiological roles in tuberculosis, and several inhibitor classes have shown enzyme inhibition and growth effects in mycobacterial systems.^33-35^ Reviews and experimental reports describe potent inhibition of β-class enzymes from *M tuberculosis* by sulfonamides such as acetazolamide and ethoxzolamide, as well as dithiocarbamates and related zinc-binding groups.^30,36,37^ Ethoxzolamide has been shown to reduce virulence-associated programs through PhoPR signaling in *M tuberculosis*, which supports the broader concept that carbonic anhydrase inhibition can sensitize bacilli to stress and to companion drugs.^38^ More recent work reported benzothiadiazinone-based inhibitors that suppress the growth of drug-resistant *M tuberculosis* strains, underscoring continued interest in carbonic anhydrase as a tractable antibacterial target.^37^ Emerging studies have also begun to explore β-class enzymes in this organism and propose them as promising targets for new mechanisms of action. The present results fit this landscape by pointing to 2 non-sulfonamide-like scaffolds that stabilize a β carbonic anhydrase model and that show activity in *M marinum*, a commonly used surrogate for tuberculosis research.^33,37^

Comparison with known compounds helps set expectations for future optimization. Acetazolamide and ethoxzolamide are classic carbonic anhydrase inhibitors with low nanomolar enzyme potency against *M tuberculosis* β-carbonic anhydrases, and they have demonstrated antibacterial activity in cell cultures.^39-41^ In this study, F2686-0257 showed growth inhibition and rifampicin potentiation in *M marinum*, supporting a carbonic anhydrase-based mechanism and indicating that further improvements in potency may enhance cellular activity. F1011-1367, in contrast, relied more on polar interactions within the binding pocket and displayed weaker inhibition, suggesting the need for structural modifications to strengthen aromatic contacts and improve stability. Thus, the results highlight β-carbonic anhydrases as relevant antibacterial targets and identify 2 chemotypes with distinct binding features that can guide scaffold optimization for future drug design.

### Limitations

This study has certain limitations that should be considered when interpreting the findings. First, the β-carbonic anhydrase structure used for computational analyses was derived from homology modeling rather than an experimentally determined structure. Although the model showed high sequence identity and favorable stereochemical quality, structural uncertainties inherent to modeled systems may influence docking and molecular dynamics outcomes.

Second, while the present work integrates extensive computational analyses with cellular growth inhibition assays, direct enzymatic inhibition and kinetic characterization against purified β-carbonic anhydrase were not included here. These biochemical studies are currently in progress using recombinant protein and will be reported separately to provide a detailed mechanistic and kinetic evaluation of target engagement.

Third, bacterial growth inhibition was assessed across multiple compound concentrations and time points under defined in vitro conditions. However, formal minimum inhibitory concentration (MIC) values were not calculated using standardized broth microdilution protocols. Therefore, the concentrations reported in this study should be interpreted as comparative indicators of growth suppression and drug-interaction trends rather than definitive MIC endpoints.

Finally, ADME and toxicity profiles were evaluated using established in silico prediction tools. While informative at the early discovery stage, these predictions require experimental pharmacokinetic and toxicological validation to fully assess translational potential.

## Conclusion

This study identified 2 β-carbonic anhydrase–targeting chemotypes with antituberculosis potential using a combined computational and experimental strategy. Homology modeling, docking, molecular dynamics, and SAR analyses revealed that F2686-0257 slightly stabilized the binding site through aromatic and hydrophobic contacts with moderate ligand motion, while F1011-1367 favored a polar interaction profile accompanied by greater reorientation within the pocket. These distinct binding behaviors were reflected in *M marinum* assays, where F2686-0257 inhibited growth at lower concentrations and enhanced rifampicin activity, whereas F1011-1367 showed weaker effects without synergy. Structural and physicochemical analyses further highlighted rigid aromatic scaffolds, appropriate molecular size, limited fluorination, and balanced polarity as desirable features for inhibition. Hence, these findings support mycobacterial carbonic anhydrases as valid antibacterial targets and provide guiding principles for the design of new scaffolds that may complement existing tuberculosis treatments.

## Supplemental Material

sj-xlsx-1-bbi-10.1177_11779322261427120 – Supplemental material for Computational and Experimental Characterization of Mycobacterium marinum β-Carbonic Anhydrase Inhibitors Supplemental material, sj-xlsx-1-bbi-10.1177_11779322261427120 for Computational and Experimental Characterization of Mycobacterium marinum β-Carbonic Anhydrase Inhibitors by Niaz Morshed, Md. Selim Reza, Ratul Bhowmik and Ashok Aspatwar in Bioinformatics and Biology Insights

sj-xlsx-2-bbi-10.1177_11779322261427120 – Supplemental material for Computational and Experimental Characterization of Mycobacterium marinum β-Carbonic Anhydrase Inhibitors Supplemental material, sj-xlsx-2-bbi-10.1177_11779322261427120 for Computational and Experimental Characterization of Mycobacterium marinum β-Carbonic Anhydrase Inhibitors by Niaz Morshed, Md. Selim Reza, Ratul Bhowmik and Ashok Aspatwar in Bioinformatics and Biology Insights

sj-xlsx-3-bbi-10.1177_11779322261427120 – Supplemental material for Computational and Experimental Characterization of Mycobacterium marinum β-Carbonic Anhydrase Inhibitors Supplemental material, sj-xlsx-3-bbi-10.1177_11779322261427120 for Computational and Experimental Characterization of Mycobacterium marinum β-Carbonic Anhydrase Inhibitors by Niaz Morshed, Md. Selim Reza, Ratul Bhowmik and Ashok Aspatwar in Bioinformatics and Biology Insights
